# Cultivar-Dependent Variation of the Cotton Rhizosphere and Endosphere Microbiome Under Field Conditions

**DOI:** 10.3389/fpls.2019.01659

**Published:** 2019-12-20

**Authors:** Feng Wei, Lihong Zhao, Xiangming Xu, Hongjie Feng, Yongqiang Shi, Greg Deakin, Zili Feng, Heqin Zhu

**Affiliations:** ^1^Zhengzhou Research Base, State Key Laboratory of Cotton Biology, Zhengzhou University, Zhengzhou, China; ^2^Institute of Cotton Research, Chinese Academy of Agricultural Sciences, Anyang, China; ^3^NIAB East Malling Research, East Malling, West Malling, Kent, United Kingdom

**Keywords:** cotton, *Verticillium dahliae*, wilt resistance, rhizosphere microbiome, root endosphere

## Abstract

*Verticillium* wilt caused by *Verticillium dahliae* is a common soil-borne disease worldwide, affecting many economically important crop species. Soil microbes can influence plant disease development. We investigated rhizosphere and endosphere microbiomes in relation to cotton cultivars with differential susceptibility to *Verticillium* wilt. Soil samples from nine cotton cultivars were assessed for the density of *V. dahliae* microsclerotia; plants were assessed for disease development. We used amplicon sequencing to profile both bacterial and fungal communities. Unlike wilt severity, wilt inoculum density did not differ significantly among resistant and susceptible cultivars. Overall, there were no significant association of alpha diversity indices with wilt susceptibility. In contrast, there were clear differences in the overall rhizosphere and endosphere microbial communities, particularly bacteria, between resistant and susceptible cultivars. Many rhizosphere and endosphere microbial groups differed in their relative abundance between resistant and susceptible cultivars. These operational taxonomic units included several well-known taxonomy groups containing beneficial microbes, such as Bacillales, Pseudomonadales, Rhizobiales, and *Trichoderma*, which were higher in their relative abundance in resistant cultivars. Greenhouse studies with sterilized soil supported that beneficial microbes in the rhizosphere contribute to reduced wilt development. These findings suggested that specific rhizosphere and endosphere microbes may contribute to cotton resistance to *V. dahliae*.

## Introduction

The plant microbiome, referred to as the host's second genome, comprises diverse microbial classes. Plant root-associated microbiomes represent a huge reservoir of biological diversity, in the order of tens of thousands of species (Berendsen et al., 2012). Plants depend upon beneficial interactions between roots and microbes for nutrient uptake, and improved tolerance to biotic and abiotic stress (Mendes et al., 2011; Berendsen et al., 2012; Zhang et al., 2019). Beneficial soil microbes contribute to pathogen resistance (Mendes et al., 2011; Berendsen et al., 2012), drought tolerance (Lau and Lennon, 2012), and promoting plant growth (Pii et al., 2015). In return, plants secrete up to 20% of their fixed carbon and 15% of their nitrogen into the rhizosphere, thus supporting microbial communities (Sasse et al., 2018).

Maintenance of a diverse population of soil microorganisms is crucial in achieving sustainable agriculture. Interactions between microbiota and their host plants have recently received much attention, mainly due to advances in sequencing technology. Microbial composition has been investigated in various plant species, such as *Arabidopsis* (Bulgarelli et al., 2012; Lundberg et al., 2012; Schlaeppi et al., 2014; Duran et al., 2018), *Populus* (Gottel et al., 2011; Beckers et al., 2017), maize (Peiffer et al., 2013), and rice (Edwards et al., 2015; Zhang et al., 2019). Characterization of the core root microbiome of *Arabidopsis* showed that the dominant phyla in the endosphere were much less diverse than in the rhizosphere (Bulgarelli et al., 2012; Lundberg et al., 2012; Schlaeppi et al., 2014). In field conditions, geographical location was the factor explaining most of the variability in the root-associated microbiomes of maize (Peiffer et al., 2013), poplar (Shakya et al., 2013), and rice (Edwards et al., 2015), whilst plant genotypes accounted for a smaller but still significant proportion of the variability (Peiffer et al., 2013). It has been suggested that plants may assemble their microbiomes in two steps; (1) a general recruitment of microbes to the vicinity of the root, and (2) genetic filtering processes that allows specific microbes into roots (Bulgarelli et al., 2013).

Recent evidence suggests that variability in plant genotypes, even at a single gene locus, can have a significant impact on rhizosphere microbiomes (Stringlis et al., 2018; Zhang et al., 2019). Weinert et al. (2011) detected 2432 bacterial operational taxonomic units (OTUs) in the potato rhizosphere, of which 40% had a site-specific abundance, 9% had a cultivar-dependent abundance at the one or the other field site, and 4% at both sites. Interestingly, OTUs which differed in relative abundance among three studied potato cultivars mainly belonged to groups known to contain isolates with biocontrol potential, such as the Pseudomonales, Streptomycetaceae, and Micromonosporaceae (Weinert et al., 2011). Thus, specific plant genotypes may recruit beneficial microorganisms that help them defend against pathogens. Specific microbial species in soil are associated with soil suppressiveness of pathogens. For instance, soil suppression of wheat soil-borne pathogens was ascribed to the differences in the ability of wheat cultivars to accumulate naturally occurring DAPG-producing *Pseudomonas* spp. (Meyer et al., 2010). Similarly, the amount of antibiotics produced by specific microbial biocontrol strains in the rhizosphere differed between wheat cultivars (Okubara and Bonsall, 2008) and cultivar-specific differences in the ability to accumulate naturally occurring specific biocontrol bacteria in the rhizosphere were found in Swiss winter wheat (Meyer et al., 2010).

Cotton (*Gossypium hirsutum* L.) is an important commercial crop grown worldwide. *Verticillium* wilt, caused by the soil-borne fungal pathogen *Verticillium dahliae* Kleb., is a major disease of cotton. The primary inoculum of *V. dahliae* is microsclerotia, fungal resting structures, in dead plant tissues and in soil. Microsclerotia may survive in soil for more than 10 years in the absence of a host (Pegg and Brady, 2002). Chemical fumigation has been an indispensable tool for controlling soil-borne pathogens; however, several fumigants have already been banned or face an uncertain future due to legislation (Martin, 2003). Cultivars differ in their susceptibility to *V. dahliae*. Threshold values of 4.0 and 7.0 V*. dahliae* CFU g^−1^ soil are needed for infecting susceptible and resistant cultivars, respectively (Wei et al., 2015). It is unclear whether and, if so, how cultivar resistance against *V. dahliae* is related to rhizosphere microbes and root endophytes.

In the present study, we used amplicon sequencing to characterize endosphere and rhizosphere microbial communities of nine cotton cultivars with differing resistance against wilt in a designed field experiment. We established an association of specific endosphere and rhizosphere microbes with wilt resistance and to test this association we then carried out a greenhouse study in which the nine cultivars were inoculated with *V. dahliae* in sterilized field soils.

## Materials and Methods

### Field Experiment Design

A field experiment was conducted at the Institute of Cotton Research of Chinese Academy of Sciences (Anyang, China) to assess whether rhizosphere or endosphere microbial communities and specific microbial groups are associated with cultivar wilt resistance. The soil at the experimental site is classified as cambisol type soil (FAO, 1998). A completely randomized block design with three blocks was used. Nine cultivars were included: NXC1208, SNM9, ZM9421, LMY21, GXM25, BM16, JK10, KM50, and JM11. Each block consisted of nine plots, each was 5 m long with two rows (0.8 m between two rows); neighbouring plots were separated by 0.8 m. In each plot, six soil cores (three randomly selected points in each row, 2.5 cm in diameter, from just below the surface to a depth of 15 cm) were collected just before planting, and bulked into a single sample per plot for assessing wilt inoculum level. In April 2016, seeds were sown with a within-row plant-to-plant distance of 25 to 30 cm. During late August (at the boll-forming stage), approximately 16 weeks after sowing, wilt severity on all individual plants was recorded on a scale of 0 to 4: 0 = no symptoms, 1 = ≤33%, 2 = >33% and ≤66%, 3 = > 66% and ≤99%, and 4 = 100% leaves with wilt symptoms. An overall disease index (DI) was calculated for each plot:

$$DI=\frac{0\cdot n_{0}+1\cdot n_{1}+2\cdot n_{2}+3\cdot n_{3}+4\cdot n_{4}}{4\cdot n}\times 100\%$$

Where *n*_0_–*n*_4_ was the number of plants with the corresponding disease ratings (0–4), and *n* was the total number of plants assessed in each plot.

In order to ascertain that wilt development of individual plants (hence cultivar resistance) was not mainly due to the level of *V*. *dahliae* inoculum, we estimated the density of *V*. *dahliae* inoculum using a wet sieving and plating method (Wei et al., 2015). Then ANOVA was applied to assess when differences in inoculum densities could largely account for cultivar differences in the observed wilt severities.

### Sample Collection of Rhizosphere and Endosphere Fractions

#### Rhizosphere Samples Collection

At the same time as wilt assessment, three plants from each plot were randomly selected and carefully removed from the soil using a spade. Root systems of the three plants from each plot were first vigorously shaken to remove loosely adhering soil particles, then the root systems were combined as a single composite sample. Plant fine roots were cut into pieces of approximately 2 cm length using sterile scissors. Rhizosphere samples were harvested in aliquots of 20 g roots in 500 ml screw-cap bottles. Each bottle was filled up to 300 ml with 1:50 TE buffer (1 M Tris, 500 mM EDTA, and 1.2% Triton diluted in sterile distilled water) and shaken at 270 rpm for 1 h (room temperature). The root-washing suspension was filtered with sterile cheesecloth and centrifuged (4,000 × *g*) at 4°C for 20 min (Wei et al., 2016a). The supernatant was discarded by pipetting. This step was repeated several times before the pellets were re-suspended in remaining solution, transferred to a 2 ml Eppendorf tube and centrifuged at 14,000 × *g* for 20 min. The pellets were immediately frozen and stored at −80°C before DNA extraction.

#### Endosphere Samples Collection

After washing, clean roots were moved to a new bottle and surface sterilized as described in Li et al. (2010). Cotton root samples that were not contaminated as determined by a culture-dependent disinfection test (Li et al., 2010) were used for subsequent analyses. Roots were cut into pieces of approximately 1 cm in length using sterile scissors and homogenized with a soft-headed hammer as described earlier by Hardoim et al. (2011) in a sterile polythene bag to release endophytes. The residues were shaken again with glass beads in 300 ml 1:50 TE buffer for 3 h at room temperature to detach microorganisms (Kadivar and Stapleton, 2003). The washing suspension was filtered with sterile cheesecloth. To collect root endosphere microbes, the suspension was centrifuged (4,000 × *g*) at 4˚C for 20 min and the supernatant discarded. This step was repeated several times and the pellets were re-suspended in the remaining solution, transferred to a 2 ml Eppendorf tube and centrifuged at 14,000 × *g* for 20 min. The pellets were immediately frozen and stored at −80°C until DNA extraction.

### DNA Extraction and Next-Generation Sequencing

Extraction of DNA and next-generation sequencing for rhizosphere and endosphere samples followed the same procedure.

Cells (250 mg) were re-suspended in 500 µl MoBio PowerSoil bead solution, and DNA was extracted using the MoBio PowerSoil DNA Kit (MoBio Laboratories, Carlsbad, CA, USA) following the manufacturer's protocol; extractions were carried out in triplicate for each sample, pooled after extraction, and quantified using a NanoDrop ND-2000 spectrophotometer (NanoDrop Technologies, Wilmington, DE, USA).

For bacteria, the V5-V7 16S rRNA gene region was amplified in triplicates for each sample using the 799F (Chelius and Triplett, 2001) and 1193R primers (Bodenhausen et al., 2013) with the barcodes. For fungi, primers ITS5 and ITS2 (White et al., 1990) with the barcodes were used to amplify the ITS1 region. For amplification, the 30 μl reaction mixtures contained 15 μl of Phusion^®^ High-Fidelity PCR Master Mix (New England Biolabs), 0.2 μM of forward and reverse primers, and 10 ng template DNA. PCR amplification was performed using a Bio-Rad T100™ thermal cycler (Hercules, CA, USA) with the following amplification cycles: 98°C for 1 min, followed by 30 cycles of denaturation at 98°C for 10 s, annealing at 50°C for 30 s, and elongation at 72°C for 30 s; Finally 72°C for 5 min. Negative and positive controls were included in all amplifications.

The PCR products were mixed with the same volume of 1× loading buffer containing SYBR green (Takara Biotechnology Co., Ltd) and electrophoresed on 2% agarose gel for confirmation. PCR products from three technical replicates were mixed in equidensity ratios. Then, the mixed PCR products were purified with the GeneJET Gel Extraction Kit (Thermo Scientific, Fermentas, USA). Sequencing libraries were generated using TruSeq^®^ DNA PCR-Free Sample Preparation Kit following manufacturer's recommendations and index codes were added. The library quality was assessed on the Qubit 2.0 Fluorometer (Life Technologies, USA) and Agilent Bioanalyzer 2100 system. Finally, the library was sequenced on an Illumina HiSeq 2,500 and 250 nucleotide paired-end reads were generated. All samples were sequenced in one run: total 108 samples—54 samples (nine cotton cultivars × three replicates × two niches) for 16S rRNA gene sequences and 54 samples (nine cotton cultivars × three replicates × two niches) for ITS sequences.

### Sequence Processing

High-quality sequences were obtained for rhizosphere and endosphere samples by quality control and filtering of sequence quality with stringent criteria following our previous publication (Tilston et al., 2018) and was carried out separately for the four type of data sets (16S and ITS for endosphere and rhizosphere). High quality sequences were first dereplicated and unique sequences with only one read were discarded. Then, all unique sequence reads were sorted by their respective frequencies and clustered into operational taxonomic units (OTUs) at 97% similarity with a representative sequence generated for each OTU. All OTU processing was carried out with the UPARSE pipeline (Version 10.0) (Edgar, 2013) unless specified otherwise. The clustering algorithm also removed chimeras. The SINTAX algorithm (https://www.drive5.com/usearch/manual/sintax_algo.html) then assigned each OTU representative sequence to taxonomic ranks by alignment with the gene sequences against two reference databases: Unite V7 fungal database (Kõljalg et al., 2013) and RDP training set 15 bacterial database (Cole et al., 2014). Finally, an OTU table (a sample-by-observation contingency table) was generated by aligning all sequences filtered with far less stringent criteria with the OTU representative sequences as described by Deakin et al. (2018).

### Statistical Analysis of Sequence Data

Alpha diversity were calculated by analysing the observed OTUs, Chao1, Shannon, and Simpson indices using the R vegan 2.3-1 package (Dixon, 2003). The rank of alpha diversity indices were subjected to ANOVA to assess the differences between wilt susceptible and resistant cultivars *via* a permutation of significance.

To assess differences in the overall microbial communities among cultivars and between wilt resistant/susceptible cultivars (i.e. beta diversity), we used two approaches. First, UniFrac distances between samples were calculated, subjected to non-dimensional scaling analysis, and analyzed with permutation multivariate ANOVA (PERMANOVA). In this analysis, library size normalization was performed using the median-of-ratios method implemented in DESeq2 (Anders and Huber, 2010; Love et al., 2014). Second, principal component analysis (PCA) were applied to the library size normalized reads using the DESeq2 variance stabilization transformation (VST). ANOVA was then performed to assess the difference between wilt susceptible and resistant cultivars as well as between all cultivars on the first four PC scores.

Once we had tested whether there was an overall association of both endosphere and rhizosphere microbial communities with cultivar wilt resistance, we then conducted further analysis to identify specific (or core) microbes that differed significantly in their relative abundances between wilt susceptible and resistant cultivars. For this purpose, DESeq2 was applied to normalized OTU count data without rarefaction (McMurdie and Holmes, 2013). DESeq2 also implements an algorithm for the automatic filtering of OTUs before differential abundance analysis using several criteria, including variance in abundance across samples and overall abundance level. To correct for the false discovery rate associated with multiple testing, the Benjamini-Hochberg (BH) adjustment was used with DESeq2 (Benjamin and Aikman, 1995). For tree view graphs, OTU abundances were aggregated at each taxonomic rank (at the SINTAX confidence of 0.8) and these aggregated count values were tested for differential abundance between wilt susceptible and tolerant cultivars with DESeq2 as above.

### Greenhouse Trials

To exclude the effects of rhizosphere microbes on wilt development, the nine cultivars were assessed for wilt in sterilized soil in a greenhouse. Before planting, soil to a depth of 20 cm from the field experimental site was collected autoclaved at 121°C and 115 kPa twice, each for 45 min. A sterility check using plating method (Trevors, 1996) was implemented to ensure that the sterilization process was successful. Cultures of *V*. *dahliae* Vd076 isolate in the maize-sand (V/V = 1:1) medium were ground into particles, size range between 1 and 2 mm and mixed with the sterilized soil (V/V = 0.006:1). For each cultivar, there were six pots (diameter 39 cm and height 30 cm), each with five seedlings; all individual pots were located in randomized positions in the greenhouse trial area. Disease severity on individual plants was recorded eight weeks after planting using the same wilt assessment and disease indices as for the field experiment. The experiment was repeated twice. Analysis of variance (ANOVA) was applied to the disease indices; no transformation was needed to satisfy analysis assumptions. For the field data, CFU data were also included as a covariate.

## Results

### Field Disease Development

**Figure 1:**
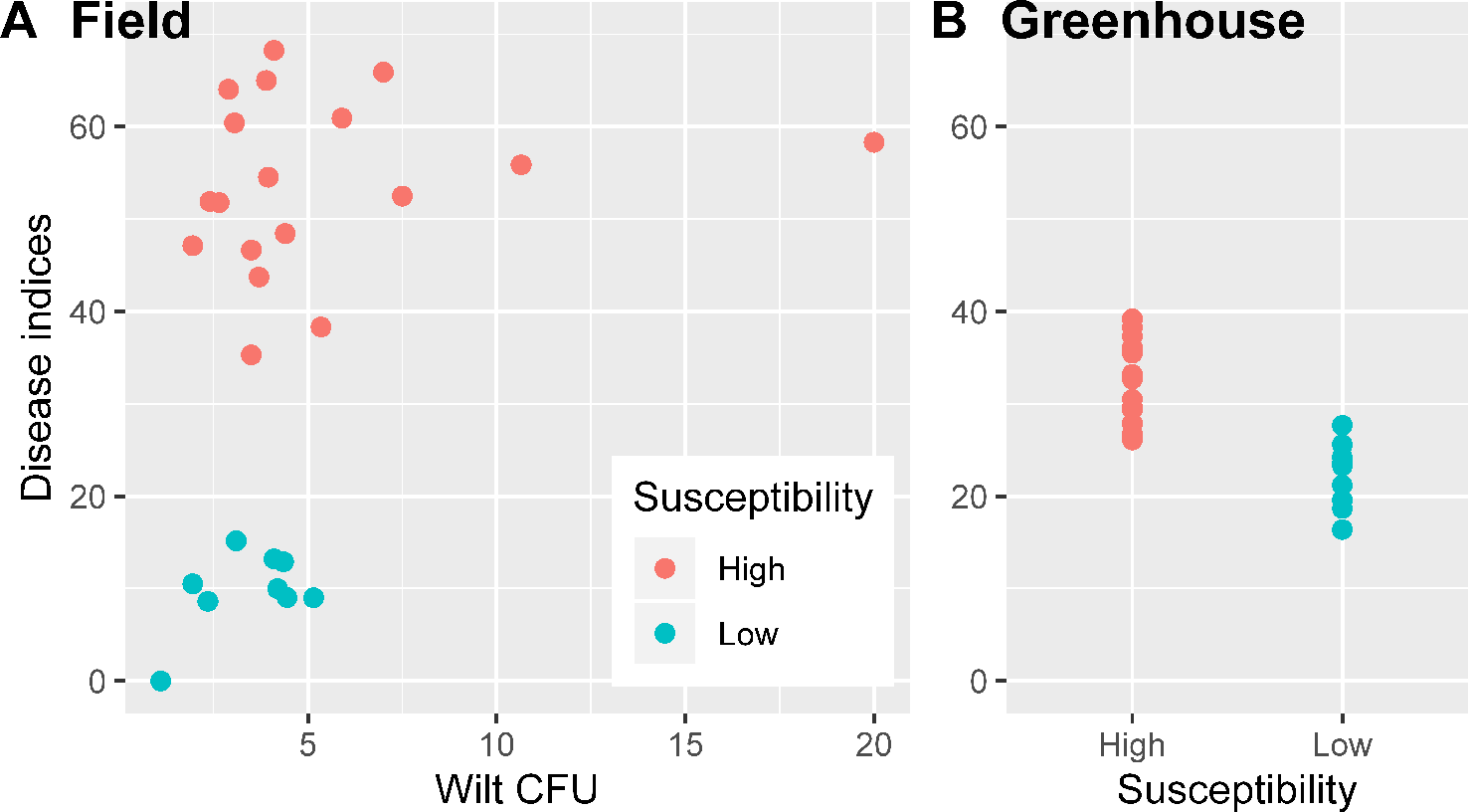
Wilt inoculum CFU values and disease indices for nine cotton cultivars in the field experiment were the averages over three replicates plots **(A)**; wilt disease indices of the greenhouse inoculation trials in sterilized soils (inoculated *Verticillium dahliae*) were summarized over three replicates experiments **(B)**.

Average wilt index ranged from 7.9 (cv. NXC1208) to 60.6 (cv. JM11) in the field and from 19.8 (cv. NXC1208) to 36.9 (cv. JM11) in the greenhouse trials (**Table 1**). Three cultivars (NXC1208, SNM9, and ZM9421) had very low wilt indices in the field trial and were classified as wilt resistant cultivars; the other six cultivars (LMY21, GXM25, BM16, JK10, KM50, and JM11) were classified as susceptible to *V*. *dahliae*. There were no significant differences in the *V*. *dahliae* CFU per gram of dried soil between the wilt resistant and susceptible cultivars in the field trial (**Figure 1A**). The high average CFU value for A59 is due to one extreme high count of 20, compared to the next highest value of 10.7. When used as a covariate in the ANOVA of individual plant data, CFU was positively related (*P* < 0.001) to wilt indices but only accounted for 7.7% of the total variance in the wilt index. In contrast, cultivar differences in the field trial accounted for 81.5% of the total variability, most (93.0%) of which were due to the differences between susceptible and resistant cultivars.

**Table 1:** *Verticillium* wilt summary of those nine cultivars included in the study; wilt inoculum CFU values and disease indices in the field experiment were the averages over three replicates plots; wilt disease indices of the greenhouse inoculation trials in sterilized soils (inoculated *Verticillium dahliae*) were summarized over three replicates experiments.

Cultivar		Field CFU (g^−1^ dry soil)	Wilt disease indices	
Name	Code		Field trial	Greenhouse trial
NXC1208	B13	2.18	7.9	19.8
SNM9	B9	3.77	9.8	21.4
ZM9421	B6	4.30	11.7	25.5
LMY21	LM21	4.48	43.5	30.4
GXM25	B7	5.37	46.1	29.6
BM16	A23	3.78	55.4	32.8
JK10	A59	8.43	58.1	33.7
KM50	A60	4.23	59.4	32.0
JM11	JM11	5.83	60.6	36.9

### General Sequence Data

**Figure 2:**
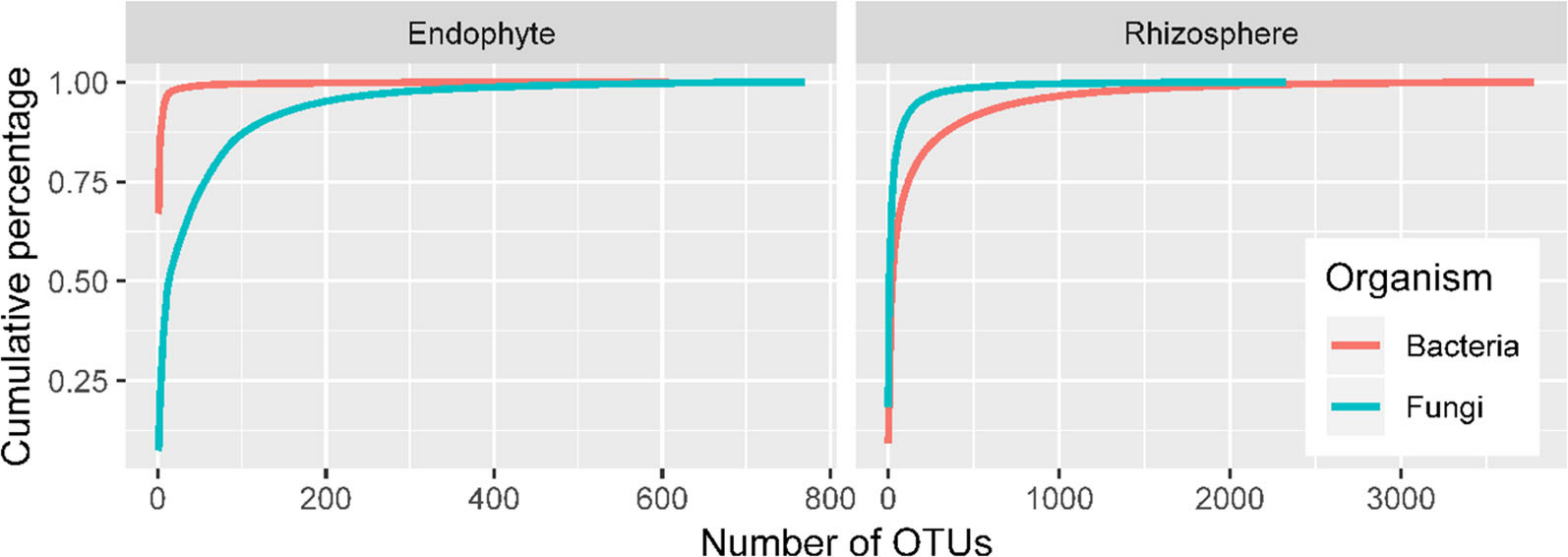
Accumulated proportion of sequence reads plotted against the number of OTUs; the OTUs were arranged in a descending order of their total counts across the 27 samples.

There were high numbers of raw sequence reads for 27 samples: ranging from 62,524 to 99,932 for 16S endophytes, from 81,614 to 99,262 for bacteria rhizosphere, from 90,406 to 108,661 for ITS endosphere, and from 78,035 to 191,678 for ITS rhizosphere. The corresponding values for the number of sequence reads included in the OTUs are from 43,518 to 81,844, from 43,839 to 67,025, from 57,702 to 96,798, and from 56,211 to 102,000. Sequencing depth is sufficient for all four combinations of ITS/16S and sample site (endosphere/rhizosphere) as shown by the rarefaction curves (**Figure 2**). Overall, a limited number of OTUs account for most sequence reads, particularly for bacterial endophytes and rhizosphere fungi (**Figure 2**). For instance, the first five bacterial OTUs in endosphere samples accounted for 90% of the total reads with the first one accounting for two thirds of the reads. The top 93 OTUs accounted for 90% of the total reads in the rhizosphere fungi.

### Taxonomy Information

**Figure 3:**
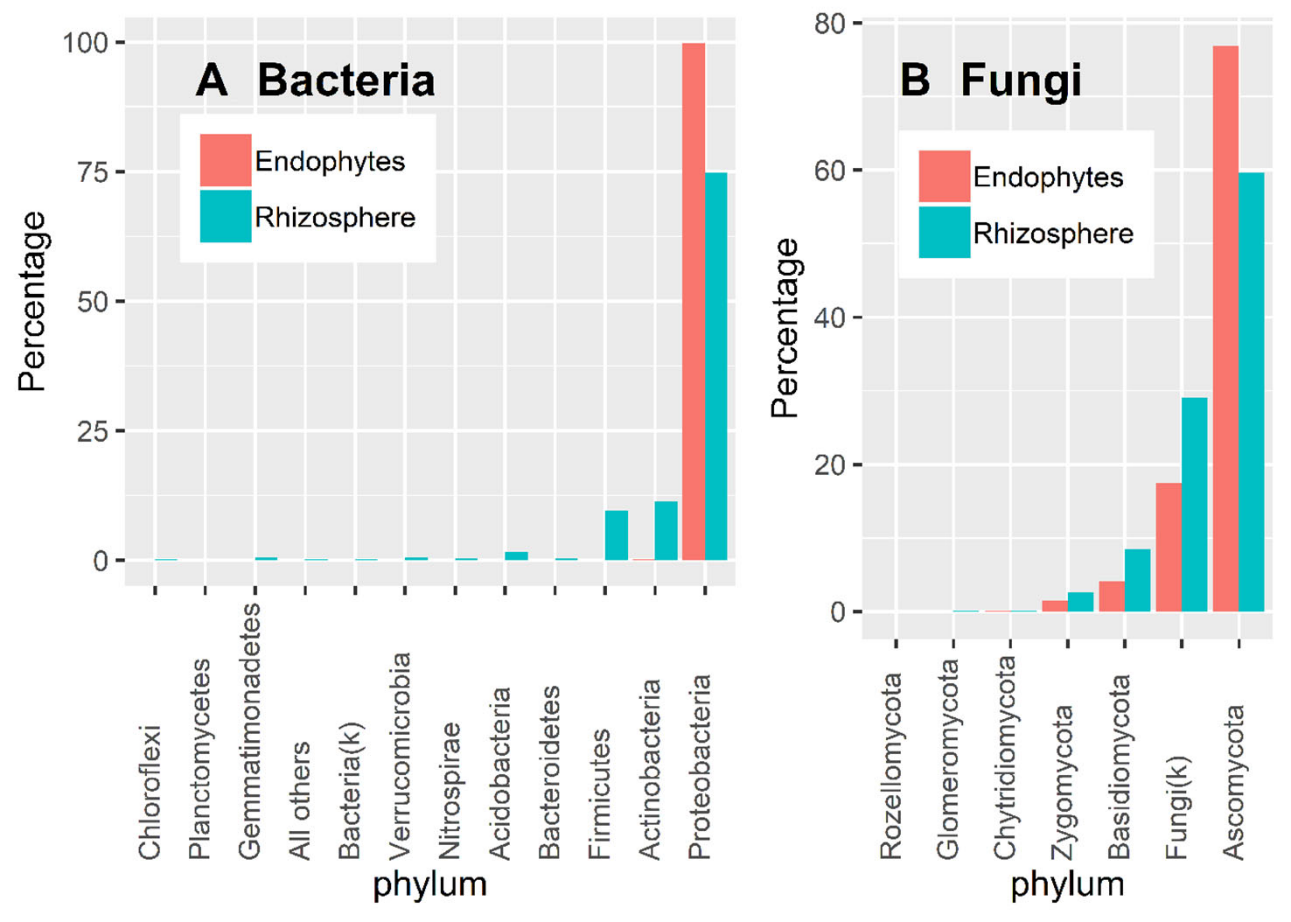
Proportion of DESeq2 normalized sequence reads assigned to different phyla at ≥ 90% confidence for bacteria **(A)** and fungi **(B)**.

Almost all 16S OTU sequences (99.9%) could be reliably assigned to the phylum level. Overall, there were more diverse bacteria in the rhizosphere than in endosphere (**Figure 3A**). Based on the DESeq2 normalized sequence data, nearly all endosphere bacterial sequences belonged to Proteobacteria (99.7%); whereas the three most abundant rhizosphere bacterial phyla were Proteobacteria (74.5%), Acidobacteria (11.4%), and Firmicutes (9.6%) (**Figure 3A**).

There is considerable numbers of fungal sequences that could not be reliably classified at the phylum level: 29.1% and 17.5% for endosphere and rhizosphere samples, respectively (**Figure 3B**). In addition to the unidentified sequences, the three most abundant endophyte fungal phyla were Ascomycota (76.9%), Basidiomycota (4.1%), and Zygomycota (1.5%); the same was true for rhizosphere samples with the corresponding values of 59.6%, 8.4% and 2.6% (**Figure 3B**). Glomeromycota only accounted for 0.01% and 0.06% of the total endosphere and rhizosphere sequence reads.

### Alpha Diversity

The number of observed bacterial OTUs was much greater for the rhizosphere samples (1,850–2,300 per sample) than for the endosphere samples (80–500 per sample). Both Simpson and Shannon indices were much higher for the rhizosphere than for the endosphere samples (**Supplementary Figures S1** and **S2**). Of the six alpha diversity indices (Chao1, Simpson, and Shannon for endosphere and rhizosphere), only the Chao1 for endosphere was greater (*P* < 0.01) for the resistant than for the susceptible cultivars (**Supplementary Figure S2**). Similarly, the number of observed bacterial endosphere OTUs was much higher for the resistant (ca. 350) than for the susceptible (ca. 200) cultivars (**Supplementary Figure S2**).

As for bacteria, within-sample fungal population was more diverse for the rhizosphere than for endosphere samples (**Supplementary Figures S3** and **S4**) but the differences were less than for bacteria. There were no significant differences in all alpha diversity indices between the wilt resistant and susceptible cultivars.

### Sample-To-Sample (Beta Diversity) Differences and Variation in Individual OTU Abundances

Both UniFrac and PCA analyses resulted in similar results and hence only PCA results are presented. For both bacteria and fungi, the proportion of sequence reads in each phylum was very similar in the samples from the wilt resistant and susceptible cultivars. **Table 3** presents the summary results from DESeq2 analysis, assessing statistical significance of the differences in the relative abundance of individual endosphere and rhizosphere bacterial and fungal OTUs between the wilt resistant and susceptible cotton cultivars. Many OTUs were automatically filtered out before DESeq2 analysis. For example, only 1,650 of 3,768 rhizosphere bacterial OTUs were compared by DESeq2.

#### Endosphere Bacteria

**Figure 4:**
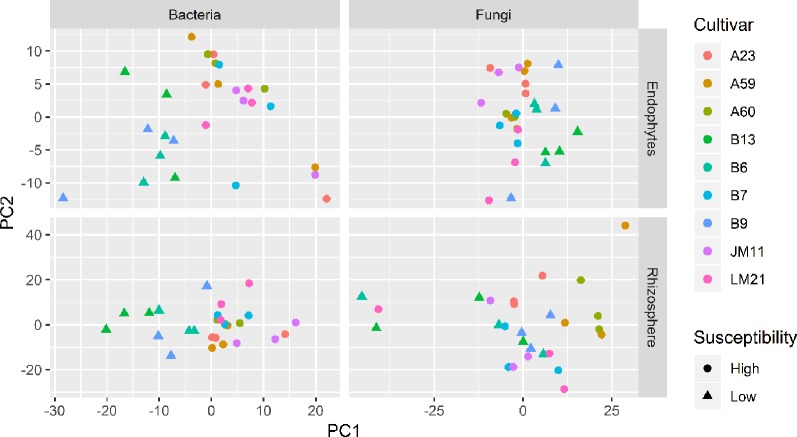
Plots of the first two principle components of normalized OTU data for bacteria and fungi in both cotton endosphere and rhizosphere of nine cultivars with differing susceptibility to *Verticillium* wilt.

**Figure 5:**
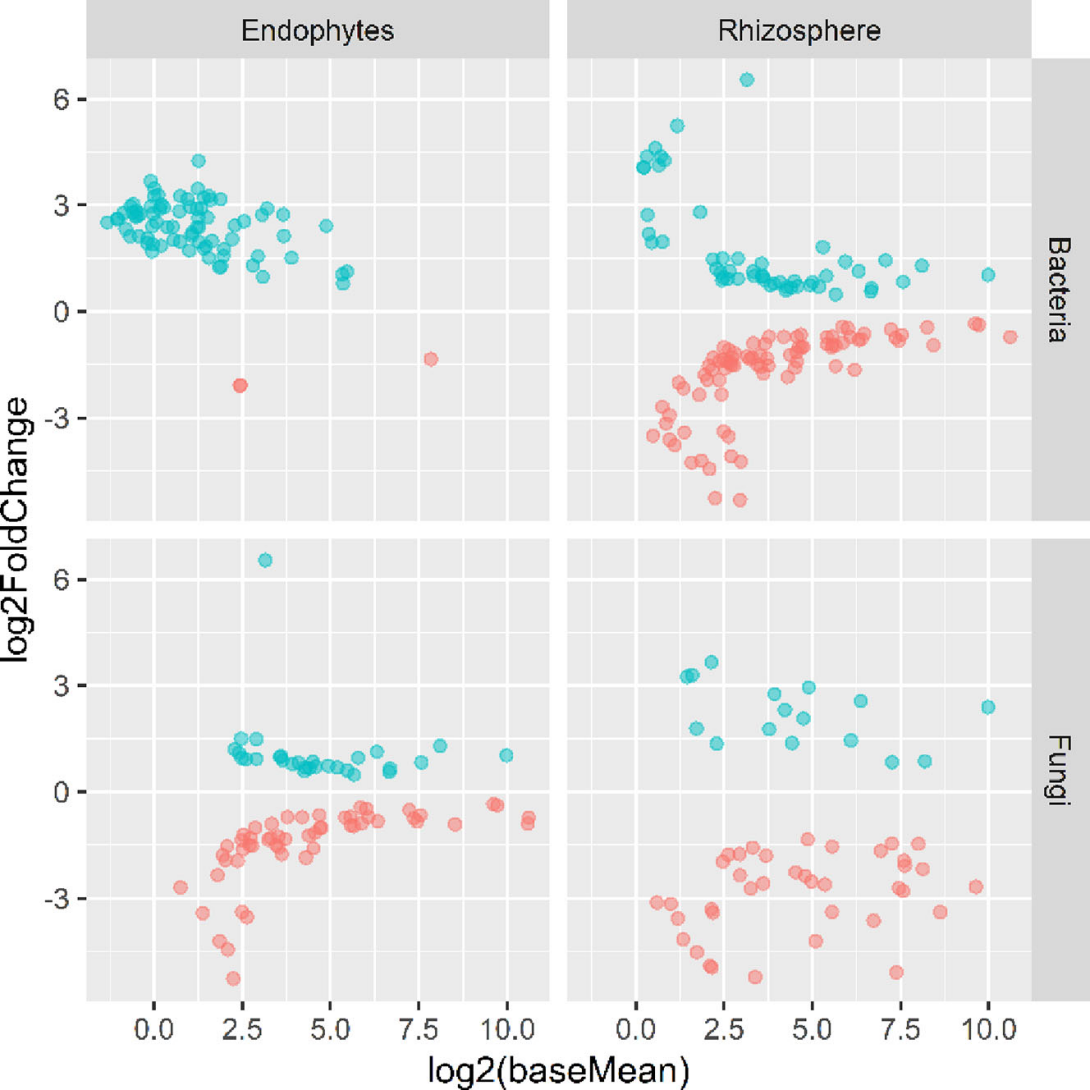
Plots of DeSeq2 analysis results for OTUs with significant (*P* < 0.05) differences between the wilt resistant and susceptible cultivars; baseMean is the average number of sequence reads for each OTU and log2FoldChange is the loge of the ratio in the number of sequence reads between the wilt resistant and susceptible cultivars. The blue symbols (positive log2FoldChange) indicates that the relative abundance of specific OTUs was greater in the wilt resistant cultivar samples than in susceptible cultivar samples.

**Figure 6:**
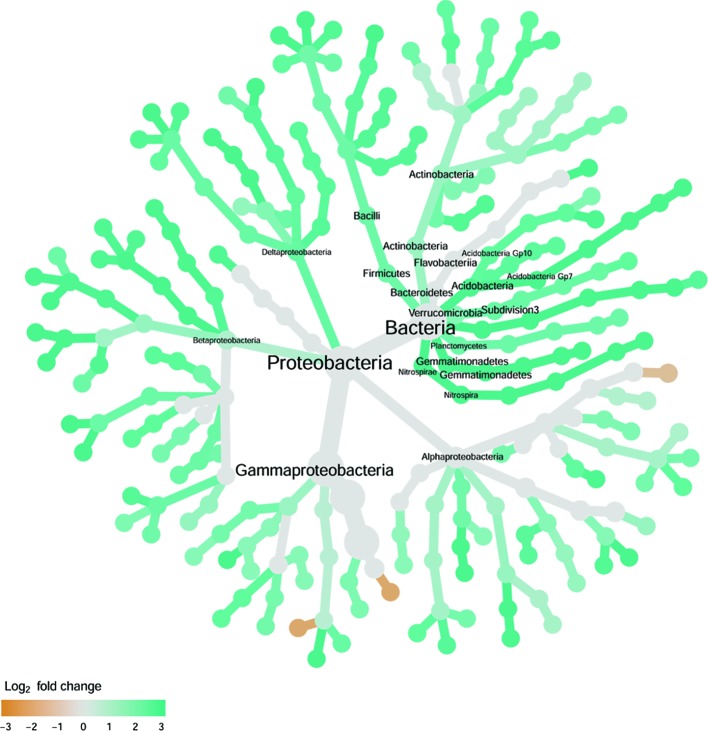
Tree views of the relative abundance between wilt-tolerant and wilt-susceptible cotton cultivars for endosphere bacteria; the relative difference in abundance is expressed as log2FoldChange with the values indicating that the relative abundance of specific OTUs was greater in the wilt tolerant cultivar samples than in susceptible cultivar samples. The size of nodes represents the abundance of endosphere bacteria at the specific taxonomic rank. Those OTUs that could not be assigned reliably to a taxonomic rank below Kingdom have been excluded from the graph. The graph is drawn with the R package—Metacoder (Foster et al., 2017).

There were significant differences between cultivars in PC1 and most of the cultivar differences in PC1 were due to the differences between the wilt resistant and susceptible cultivars (**Table 2**). The difference between the wilt resistant and susceptible was also significant for PC2. However, the wilt resistant samples were separated only along the PC1 axis (**Figure 4**) with lower PC1 scores for samples from resistant cultivars. DESeq2 analysis was applied to 313 OTUs. Wilt resistant cultivars differed in the relative abundance from susceptible cultivars for 80 OTUs; for 77 of these OTUs, resistant cultivars had higher relative abundance than susceptible cultivars (**Figure 5**, **Table 3**). Most of these OTUs cannot be assigned to the taxonomic rank below Order with confidence (**Supplementary Table S1**) and these OTUs spread across a number of bacterial classes (**Figure 6**). These 80 OTUs included one from Streptomyces, one from *Nitrospira*, eight from Bacillales (five from *Bacillus*), three from Rhodocyclaceae (one is from *Azoarcus*), one from Brevundimonas, one from *Rhodobacter*, one from *Lysobacter*, and six from Rhizobiales. Overall, average sequence counts were low except for a few OTUs (>30, **Figure 5**): two from Acidimicrobiales, one *Bacillus*, and two Rhizobiales. For one of the two Rhizobiales OTUs, susceptible cultivars had greater relative abundance than resistant cultivars.

**Table 2:** Percent variance in the first four principal components accounted by cultivars and the comparison between susceptible and resistant cultivars.

	Endosphere bacteria			Rhizosphere bacteria			Endosphere fungi			Rhizosphere fungi		
	%Var	Cul^+^	Sus^$^	%Var	Cul	Sus	%Var	Cul	Sus	%Var	Cul	Sus
PC1	27.1	39.8**	37.3**	11.3	50.3**	38.5**	13.9	35.3	29.0**	11.9	42.3	13.4**
PC2	11.1	26.1	12.9	8.6	41.4	0.7*	11.1	50.3	7.0*	8.3	38.8	0.1
PC3	6.4	56.7**	0.2	7.2	65.5**	4.8	7.8	49.2**	0.0	6.5	59.2**	7.7*
PC4	5.6	27.9*	7.2	6.2	59.8*	3.1	7.5	67.7**	6.5*	5.8	57.2**	27.1**
All PCs		40.4	18.8		39.0	8.6		41.9	10.1		37.5	6.2

^+^Between the nine cultivars; ^$^between the wilt resistant and susceptible cultivars.

*, **: P values < 0.05 and 0.01, respectively.

**Table 3:** Summary of DESeq2 analysis results, comparing the relative abundance of individual endosphere and rhizosphere bacterial and fungal OTUs between the wilt resistant and susceptible cotton cultivars.

Organisms	Number of OTUs			
	Total	After DESeq2 filtering	Significantly different	Resistant > susceptible
Endosphere bacteria	607	313	80	77
Rhizosphere bacteria	3,768	1,650	136	52
Endosphere fungi	743	709	83	29
Rhizosphere fungi	2,286	688	54	16

#### Rhizosphere Bacteria

**Figure 7:**
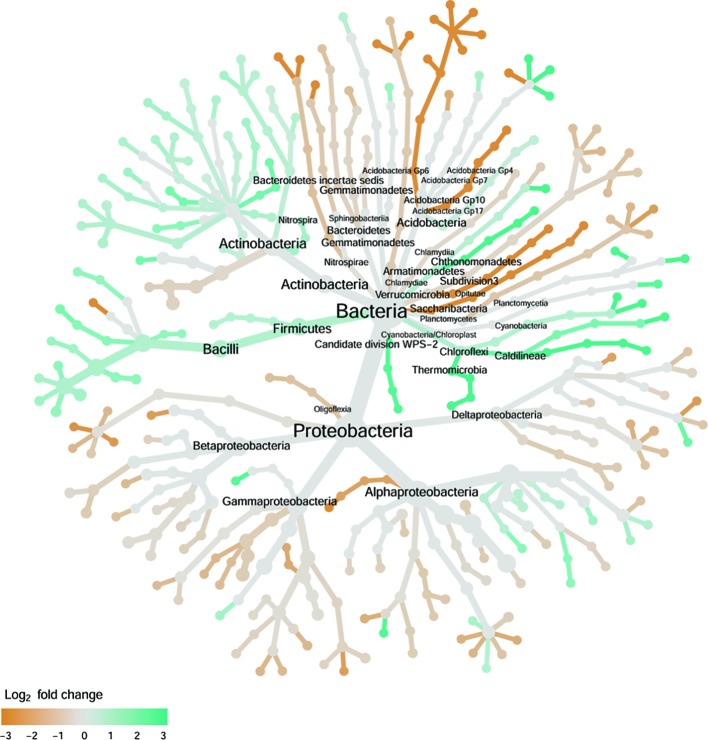
Tree views of the relative abundance between wilt-tolerant and wilt-susceptible cotton cultivars for rhizosphere bacteria; the relative difference in abundance is expressed as log2FoldChange with the values indicating that the relative abundance of specific OTUs was greater in the wilt tolerant cultivar samples than in susceptible cultivar samples. The size of nodes represents the abundance of rhizosphere bacteria at the specific taxonomic rank. Those OTUs that could not be assigned reliably to a taxonomic rank below Kingdom have been excluded from the graph. The graph is drawn with the R package—Metacoder (Foster et al., 2017).

There were significant differences between cultivars in PC1 and nearly 80% of such differences were due to the differences between the wilt resistant and susceptible cultivars (**Table 2**). Although cultivar differences were significant for PC3 and PC4 as well, there were very little differences in PC2-PC4 scores between the wilt resistant and susceptible cultivars. As for bacterial endosphere, the wilt resistant samples were separated only along the PC1 axis (**Figure 4**) with lower PC1 scores for samples from resistant cultivars. DESeq2 analysis was applied to 1,650 OTUs. For 136 OTUs, there were significant differences in the relative abundance between wilt resistant and susceptible cultivars; for 52 of these OTUs, resistant cultivars had higher relative abundance than susceptible cultivars (**Figure 5**, **Table 3**). As for rhizosphere bacteria, most of these OTUs cannot be assigned to the taxonomic rank below the order with confidence (**Supplementary Table S2**). Those OTUs with higher relative abundance in wilt tolerant cultivars were clustered within Bacilli, Actinobacteria, and Chloroflexi whereas those OTUs with lower abundance in wilt-tolerant cultivars were more spread among a number of bacterial taxa groups (**Figure 7**). Noticeable OTUs included three from Nitrospira (all Log2FoldChange < 0), two from Planctomycetaceae (all Log2FoldChange > 0), one Pseudomonadales (Log2FoldChange > 0), eight from Bacillales (seven with Log2FoldChange > 0, three from Bacillus), one from Rhodocyclaceae (Log2FoldChange < 0), seven from Rhizobiales (six with Log2FoldChange > 0), and nine Xanthomonadales (Log2FoldChange < 0). Four OTUs had very large average sequence counts (**Figure 5**): 1,561, 1,013, 843, and 784 for Ilumatobacter, *Bcaillus*, Burkholderiales, and Steroidobacter, respectively (of these four OTUs, only for *Bacillus* Log2FoldChange > 0).

#### Endosphere Fungi

**Figure 8:**
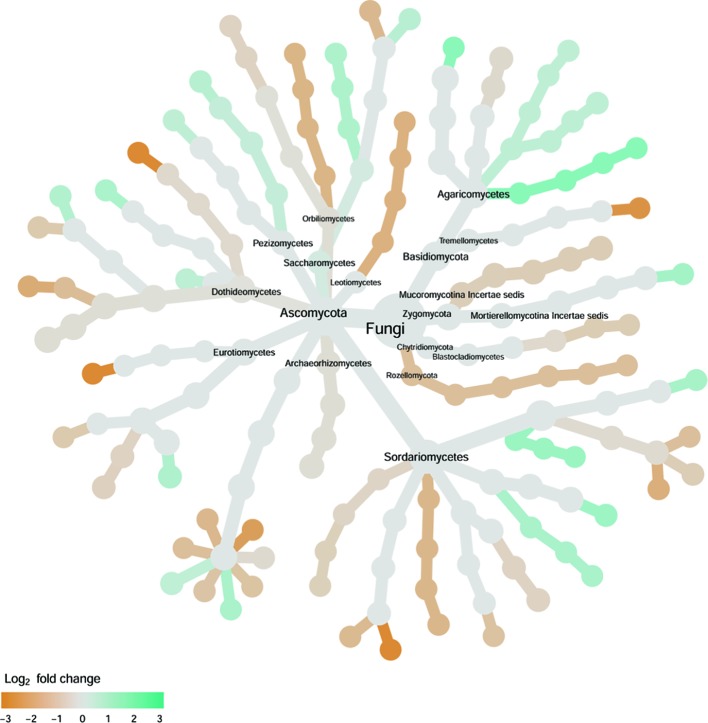
Tree views of the relative abundance between wilt-tolerant and wilt-susceptible cotton cultivars for endosphere fungi; the relative difference in abundance is expressed as log2FoldChange with the values indicating that the relative abundance of specific OTUs was greater in the wilt tolerant cultivar samples than in susceptible cultivar samples. The size of nodes represents the abundance of endosphere fungi at the specific taxonomic rank. Those OTUs that could not be assigned reliably to a taxonomic rank below Kingdom have been excluded from the graph. The graph is drawn with the R package—Metacoder (Foster et al., 2017).

Cultivars differed significantly (*P* < 0.01) in PC1 with the most (ca. 80%) of the differences attributable to the differences between the wilt resistant and susceptible cultivars (**Table 2**). The difference between the wilt resistant and susceptible was also significant for PC2 and PC4, but accounted for very low variability (**Table 2**). Samples from wilt resistant cultivars were separated from susceptible cultivars along the PC1 axis (**Figure 4**) with higher PC1 scores for resistant cultivars. DESeq2 analysis was applied to 709 OTUs. For 83 OTUs, there were significant differences in the relative abundance between wilt resistant and susceptible cultivars; for 29 of these OTUs, wilt resistant cultivars had higher relative abundance than susceptible cultivars (**Figure 5**, **Supplementary Table S3**). These 83 OTUs appear not to cluster around particular taxa groups except for several OTUs (with Log2FoldChange > 0) from Agaricomycetes (**Figure 8**). Of the 83 OTUs, 48 cannot be assigned to the taxonomic rank of phylum, and only 24 can be signed to the order rank (**Supplementary Table S3**). These 83 OTUs included *Alternaria solani*, *Aspergillus aculeatus*, *Penicillium*, *Verticillium longisporum*, and *Choanephora*; Log2FoldChange < 0 for all five OTUs except *Penicillium*. Five OTUs had very large average sequence counts (>750, **Figure 5**) but only one of them can be assigned to a rank below Kingdom, in the Pleosporaceae family (Log2FoldChange < 0).

#### Rhizosphere Fungi

**Figure 9:**
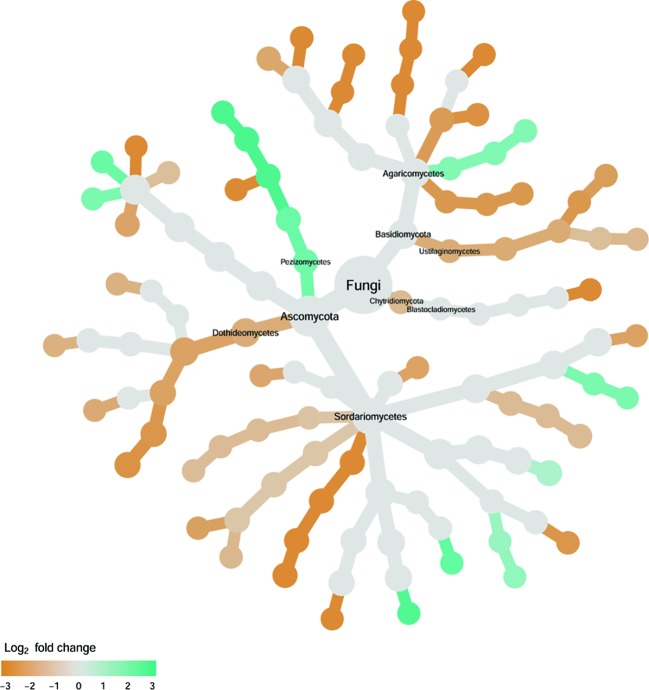
Tree views of the relative abundance between wilt-tolerant and wilt-susceptible cotton cultivars for rhizosphere fungi; the relative difference in abundance is expressed as log2FoldChange with the values indicating that the relative abundance of specific OTUs was greater in the wilt tolerant cultivar samples than in susceptible cultivar samples. The size of nodes represents the abundance of rhizosphere fungi at the specific taxonomic rank. Those OTUs that could not be assigned reliably to a taxonomic rank below Kingdom have been excluded from the graph. The graph is drawn with the R package—Metacoder (Foster et al., 2017).

Cultivar differences were only significant (*P* < 0.01) for PC3 and PC4; whereas the differences between the wilt resistant cultivars and susceptible cultivars were significant for PC1, PC3, and PC4 they did not account for much variability (**Table 2**). Samples from wilt resistant cultivars cannot be clearly separated from the susceptible cultivar samples along the PC1 or PC2 axis (**Figure 4**) although average PC1 score was lower for resistant than for susceptible cultivars. DESeq2 analysis was applied to 688 OTUs. For 54 OTUs, there were significant differences in the relative abundance between wilt resistant and susceptible cultivars; for 16 of these OTUs, wilt resistant cultivars had higher relative abundance than susceptible cultivars (**Figure 5**, **Supplementary Table S4**). As for fungal endosphere, these 54 OTUs appear not to cluster around particular taxa groups except for several OTUs (with Log2FoldChange > 0) from Pezizomycetes (**Figure 9**). Of the 54 OTUs, 33 cannot be assigned to the taxonomic rank of phylum, and a further 16 can be assigned only to the rank of family (**Supplementary Table S4**). Of the 54 OTUs, noticeable OTUs included *Alternaria*, *Trichoderma*, *Magnaporthe grisea*, *Thielaviopsis basicola*, *Microascus brevicaulis*, two from Ceratobasidiaceae, and two from Ustilaginaceae. Of these OTUS, only for *M. brevicaulis* and *Trichoderma* was Log2FoldChange > 0. Two OTUs had very large sequence counts (>790, **Figure 5**) but only one can be assigned to the taxonomy rank of Pleosporaceae (Log2FoldChange < 0).

### Wilt Development in Sterilized Soil

Although the correlation in the cultivar mean wilt indices were significant (*P* < 0.001) between field and greenhouse studies, the actual differences in the average wilt indices among the nine cultivars were much smaller in the greenhouse trial than in the field trial (**Table 1**, **Figure 1**). Average wilt index ranged from 19.8 (cv. NXC1208) to 36.9 (cv. JM11) in the greenhouse trial (**Table 1**). Cultivar differences accounted for 77.6% of the total variability, most (87.5%) of which were due to the differences between susceptible and resistant cultivars.

## Discussion

As a monocyclic disease, inoculum levels of *V. dahliae* (CFU per gram of soil) in the soil at planting plays a critical role in the development of cotton wilt (Wei et al., 2015). Increasing cultivar tolerance/resistance to *V. dahliae* leads to corresponding increases in the inoculum threshold value necessary for wilt development (Wei et al., 2015). In the present study, there were no significant differences in the *V. dahliae* CFU counts between cultivars but the wilt indices differed largely between the resistant and susceptible cultivars. Only on the basis of individual plants was the CFU positively related to observed wilt severity, but only accounted for ca. 9.0% of the total variability, compared to 81.5% by cultivars. Thus, most differences in wilt severities among cultivars in the field trial are unlikely due to the differing levels of wilt inoculum.

The most abundant bacterial rhizosphere phylum was Proteobacteria (74.5%), followed by Actinobacteria (11.4%), and Firmicutes (11.4%). Similar to studies in *Arabidopsis* (Bulgarelli et al., 2012; Lundberg et al., 2012; Schlaeppi et al., 2014) and rice (Edwards et al., 2015), the relative abundance of Proteobacteria increased in the endosphere relative to the rhizosphere, and the opposite was true for Acidobacteria and Firmicutes. With regard to fungi, Ascomycota was the most abundant phylum in both endosphere and rhizosphere. As with field strawberry plants (Wei et al., 2016b), the two most abundant fungal phyla were Ascomycota and Basidiomycota. The relative abundance of both bacterial and fungal phyla in the rhizosphere and inside roots thus appears to be similar for land plants.

Sample alpha diversity indices indicated a large reduction in the microbial diversity from rhizosphere to the root endosphere. This points to a gating role of the root surface for selective entry of bacteria and fungi into the root interior; this phenomenon has been found in other plant species, such as *Arabidopsis thaliana* (Duran et al., 2018) and rice (Edwards et al., 2015). In general, the endophytic microbiome is more specific than in rhizosphere, as fewer well-adapted bacteria are permitted to enter and survive in the plant interior (Compant et al., 2010). The complexities of specific endophyte community structures indicate that they may interact with the plant host and influence plant physiology (Gaiero et al., 2013). Specific plant factor(s) regulating endophytic communities remain little understood. A number of studies indicate that individual endophytic microbial members have antagonistic activity against pathogens (Berg et al., 2005; Procopio et al., 2009; Li et al., 2014). Such antagonistic effects may have resulted from direct effects of biologically active compounds produced by endophytes and/or indirect effects through induced resistance (Zamioudis and Pieterse, 2012).

*Verticillium* wilt resistance is mediated by quantitative trait loci and such quantitative traits can be considerably influenced by other factors, including environmental conditions and plant-associated microbiota. The composition of plant microbiome is inﬂuenced by many factors, including genotypes, plant developmental stage, and plant health (Berg et al., 2016). For instance, endophytic bacterial communities differed among cultivars (genotypes) of potato (*Solanum tuberosum* L.) (Sessitsch et al., 2004; Manter et al., 2010; Ardanov et al., 2012) and common bean (*Phaseolus vulgaris*) (de Oliveira Costa et al., 2012). This genotypic association is usually interpreted as the consequence of recruitment of specific microbes through characteristic root exudates. Structural and functional diversity of plant-associated microbiome can also greatly be affected by soil physical properties and nutrient availability (Berg and Smalla, 2009). The host-genotypic microbiome association has not yet been specifically explored in commercial agriculture.

In the present study, within-sample (alpha) diversity of nine cotton cultivars appears to have no relationships with wilt susceptibility, except bacterial endophytes: resistant cultivars appears to have more endophytic bacterial OTUs, and hence higher Chao1 (species richness) value. Similarly, a previous study reported that there were no significant differences in the abundance of isolated fungal endophytes between resistant cotton cultivars and susceptible cultivars (Li et al., 2014). However, the alpha diversity of endophytic bacteria and the abundance of culturable bacteria were both higher in the peach roots of a crown gall disease resistant cultivar then a susceptible cultivar, particularly after inoculation (Li et al., 2019).

Microbial diversity in the rhizosphere is crucial for suppressing soil-borne disease development and a higher abundance of rare species also seems to represent a barrier against soil-borne pathogens (Latz et al., 2012; van Elsas et al., 2012). Plant species and genotype are still significant factors determining composition of microbial communities resident to the rhizosphere and soils (Mazzola, 2004). We demonstrated that a large proportion of genotypic differences in plant-associated microbial community structures was associated with their resistance/susceptibility to *V. dahliae*. Present results suggest that apparent cultivar resistance to wilt may result partially from abundant beneficial microbes in the rhizosphere. A similar finding was also obtained for the association of the rhizosphere microbial community with cucumber resistance to *Fusarium* wilt (Yao and Wu, 2010). Rhizosphere microbiome structures of tomato plant differed between resistant (resistant to *Ralstonia solanacearum*) and susceptible cultivars (Kwak et al., 2018). These results suggest that plant-microbiome may contribute to the observed host resistance/susceptibility against specific pathogens.

In addition to the differences in the overall rhizosphere and endosphere microbial communities, we identified many specific bacterial and fungal groups that have differential relative abundance between wilt resistant and susceptible cotton cultivars. Although it was not possible to classify many of those groups to the rank of genus or species, most of those identified to lower taxonomic ranks appear to have plausible biological interpretations. Thus, wilt tolerance is associated with commonly known beneficial bacteria, including *Bacillus* (Egamberdieva, 2016), *Lysobacter* (Sullivan et al., 2003), Streptomyces (Niu et al., 2016), Rhizobiales (Erlacher et al., 2015) and *Pseudomonas* (Thierry et al., 2004). In addition, bacterial endophytes *Azoarcus* play an important role in N_2_-fixation in natural plant ecosystems (Franche et al., 2009). All nine Xanthomonadales were enriched in susceptible cultivars; the *Xylella* and *Xanthomonas* species in Xanthomonadales cause serious diseases in more than 400 agriculturally important plant species (Naushad and Gupta, 2013). Many fungal groups had increased relative abundance in wilt susceptible cultivars, including fungal endophytes of *Alternaria solani*, *Aspergillus aculeatus*, *V. longisporum*, *and Choanephora*, and rhizosphere fungi of *Alternaria*, *Magnaporthe grisea*, *Thielaviopsis basicola*, Ceratobasidiaceae, and Ustilaginaceae. *Alternaria* spp. and *Thielaviopsis basicola* are both known pathogens of cotton (Pullman et al., 1981; Coumans et al., 2009) and *Rhizoctonia*, of the Ceratobasidiaceae family, can cause damping-off of cotton seedlings (Pullman et al., 1981). *V. longisporum* could cause wilt diseases on cruciferous hosts (Zeise and Von Tiedemann, 2002). In wilt resistant cultivars, both *Microascus brevicaulis* and *Trichoderma* had higher abundance. *Trichoderma* spp. are the most important fungal biocontrol agents for controlling a number of plant diseases, including *Verticillium* wilt (Harman et al., 2004). In addition to being a typical soil decomposer, it is well known that *M. brevicaulis* lives within the American dog tick (*Dermacentor variabilis*); this relationship seems to be highly adapted but not as a typical host-parasite interaction. Studies have shown that *M. brevicaulis* in the form of endosymbionts exist in the host, which may provide protection against the insect-pathogenic fungus *Metarhizium anisopliae* (Yoder et al., 2008). Further research is needed to isolate and confirm which bacterial and fungal OTUs associated with *V*. *dahliae* resistance.

The greenhouse trial with sterilized soil strongly suggested that the beneficial microbes in the rhizosphere are partially responsible for reduced wilt development in the three ‘resistant cultivars’. Without these beneficial organisms, the differences between ‘resistant’ and ‘susceptible’ cultivars (as defined based on the field results) are much smaller and average cultivar wilt index is more or less in a continuum without large differences between ‘susceptible’ and ‘resistant’ cultivars. However, it was not possible to estimate possible contributions by endophytes to observed wilt differences between cultivars.

Although the use of classical single-strain biocontrol products for the management of soil-borne disease has long been a goal in commercial agriculture, there are limited examples of successful application in commercial field crop production systems (Mazzola and Freilich, 2017). Recently, there is a growing trend of designing consortia of multiple beneficial microbes for managing soil-borne diseases (Lebeis et al., 2015) and improving nitrogen use in crops (Zhang et al., 2019). The present results support this microbial consortium approach since multiple beneficial microbes were associated with wilt resistant cultivars. We have been characterizing several microbial groups (*Pseudomonas*, *Bacillus*, *Trichoderma*, etc.) for their effects on wilt development, and will further evaluate their joint effects on wilt suppression in commercial fields. Furthermore, we may need to investigate interactions of multiple cotton pathogens on wilt development since wilt susceptible cultivars are associated with high abundance of other candidate pathogens in rhizosphere and endosphere.

## Conclusion

The present study demonstrated that plant genotype contribute to the shaping of the plant-associated microbial community and specific groups of rhizosphere microbiota and root endophytes may associate with cotton resistance to *V. dahliae* when sampled at the boll-forming stage. Such an association is stronger for bacteria than for fungi. Many individual microbial OTUs differ in their relative abundance between wilt resistant and susceptible cultivars. These OTUs included several well-known taxonomy groups containing beneficial microbes, such as Bacillales, Pseudomonadales, Rhizobiales, and *Trichoderma*, with higher relative abundance associated with resistant cultivars. Greenhouse data supported that beneficial microbes in rhizosphere contribute to reduced wilt development. These findings suggested that specific rhizosphere and endosphere microbes may contribute to cotton resistance to *V. dahliae*.

## Data Availability Statement

Raw sequence data reported in this paper have been deposited (PRJEB32779) in the European Nucleotide Archive (ENA).

## Author Contributions

FW, XX, ZF, and HZ planned and designed the research and experiments. FW, LZ, HF, YS and ZF performed the experiments. FW, XX, and GD analyzed the data. FW and XX wrote the manuscript. FW and HZ acquired the funds for the study. All authors read and approved the final manuscript.

## Funding

This work was supported by National Natural Science Foundation of China (Grant No. 31901938) and the National Key Research and Development Program of China (Grant No. 2017YFD0201900).

## Conflict of Interest

The authors declare that the research was conducted in the absence of any commercial or financial relationships that could be construed as a potential conflict of interest.

## Supplementary Material

Figure S1 Alpha diversity for rhizosphere bacteria of nine cotton cultivars, classified into wilt tolerant or susceptible groups. Permutation ANOVA was applied to assess the differences in Chao1, Simpson and Shannon indices between the wilt-tolerant and susceptible cultivars.

Figure S2 Alpha diversity for endosphere bacteria of nine cotton cultivars, classified into wilt tolerant or susceptible groups. Permutation ANOVA was applied to assess the differences in Chao1, Simpson and Shannon indices between the wilt-tolerant and susceptible cultivars.

Figure S3 Alpha diversity for endosphere fungi of nine cotton cultivars, classified into wilt tolerant or susceptible groups. Permutation ANOVA was applied to assess the differences in Chao1, Simpson and Shannon indices between the wilt-tolerant and susceptible cultivars.

Figure S4 Alpha diversity for rhizosphere fungi of nine cotton cultivars, classified into wilt tolerant or susceptible groups. Permutation ANOVA was applied to assess the differences in Chao1, Simpson and Shannon indices between the wilt-tolerant and susceptible cultivars.
