# Supplementary figures and images for: Cultivar-Dependent Variation of the Cotton Rhizosphere and Endosphere Microbiome Under Field Conditions
Source: Front Plant Sci. 2019 Dec 20;10:1659. doi: 10.3389/fpls.2019.01659 (PMC6933020; doi:10.3389/fpls.2019.01659)

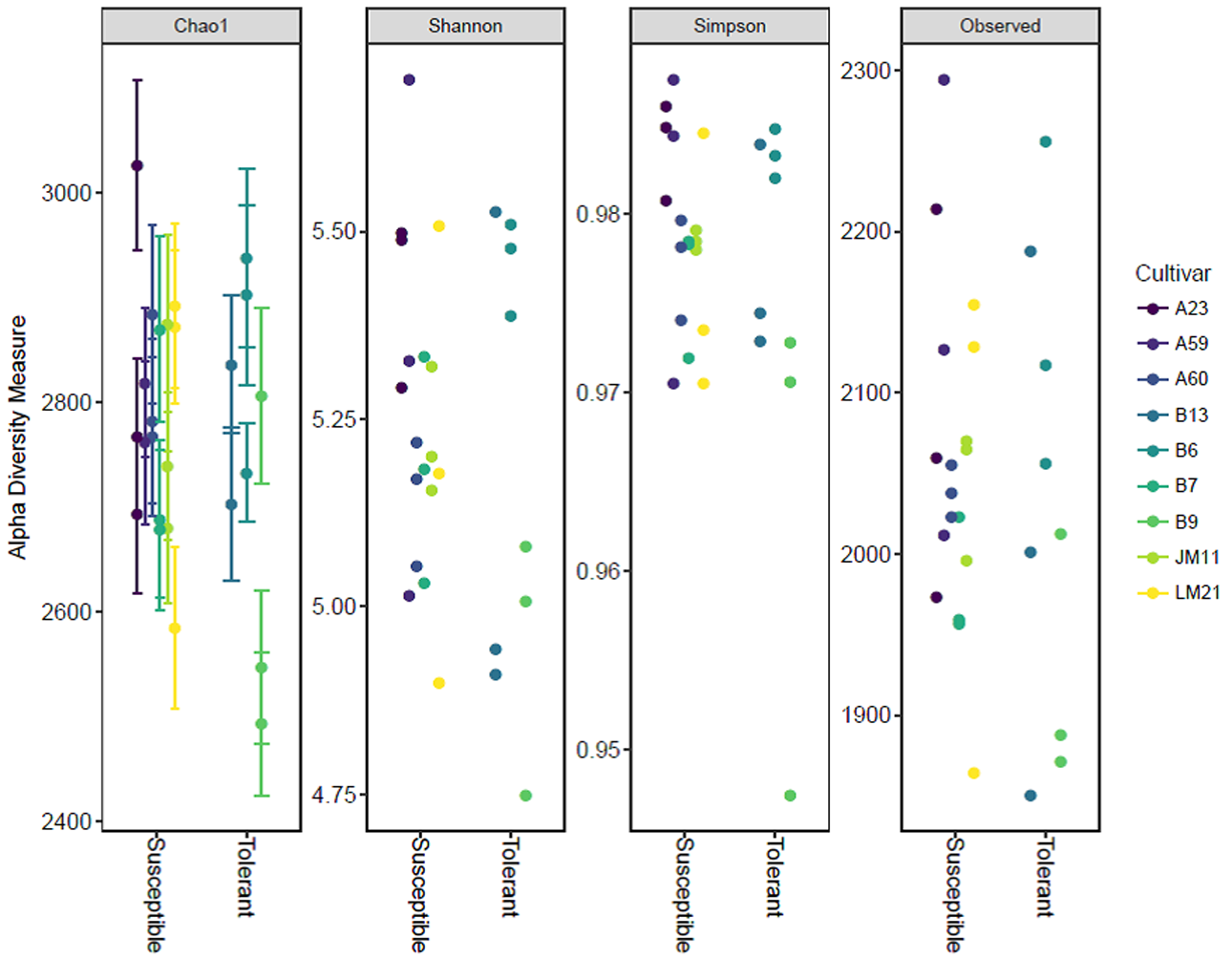

Supplement: Figure S1 — Alpha diversity for rhizosphere bacteria of nine cotton cultivars, classified into wilt tolerant or susceptible groups. Permutation ANOVA was applied to assess the differences in Chao1, Simpson and Shannon indices between the wilt-tolerant and susceptible cultivars. [file Image_1.tif]

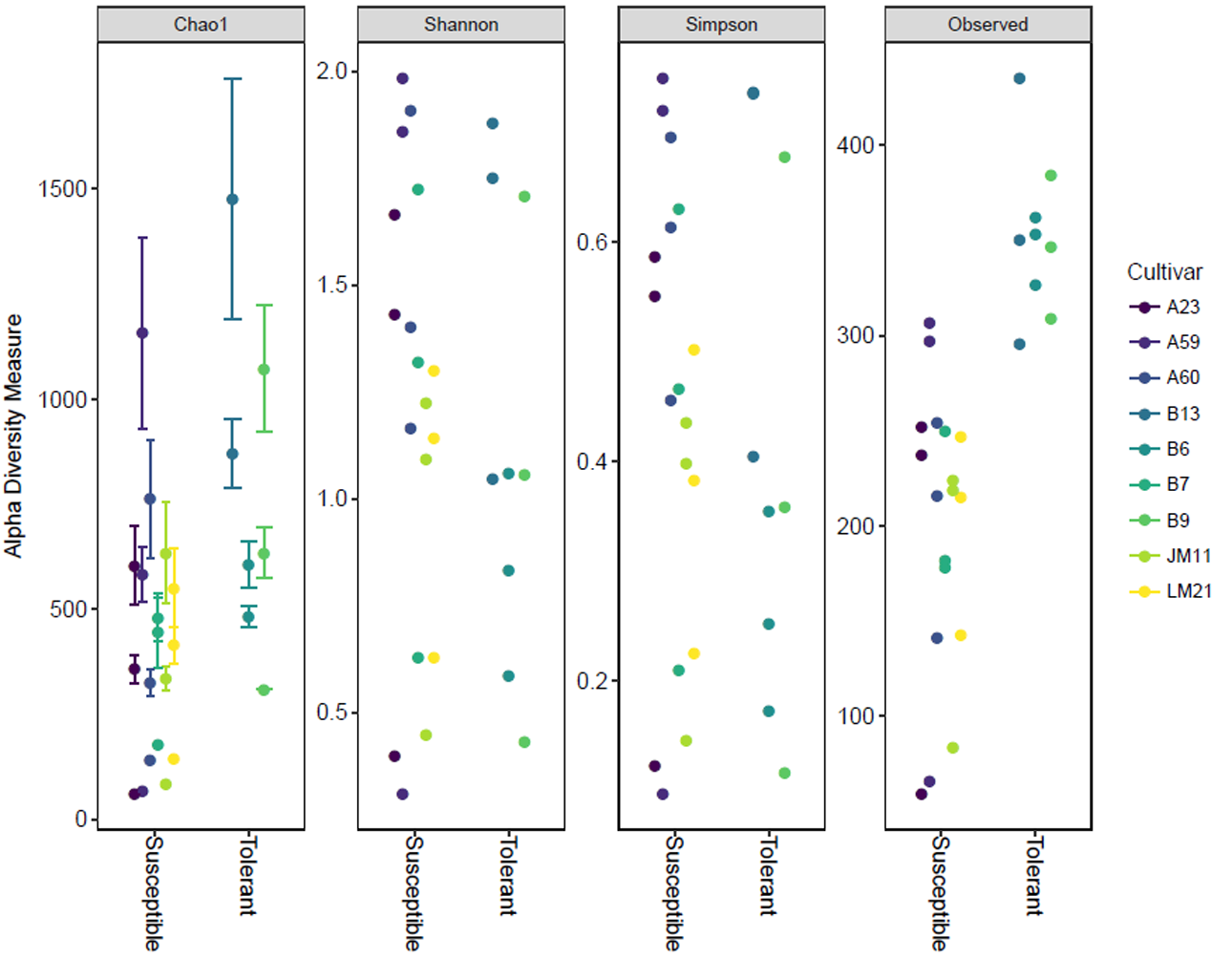

Supplement: Figure S2 — Alpha diversity for endosphere bacteria of nine cotton cultivars, classified into wilt tolerant or susceptible groups. Permutation ANOVA was applied to assess the differences in Chao1, Simpson and Shannon indices between the wilt-tolerant and susceptible cultivars. [file Image_2.tif]

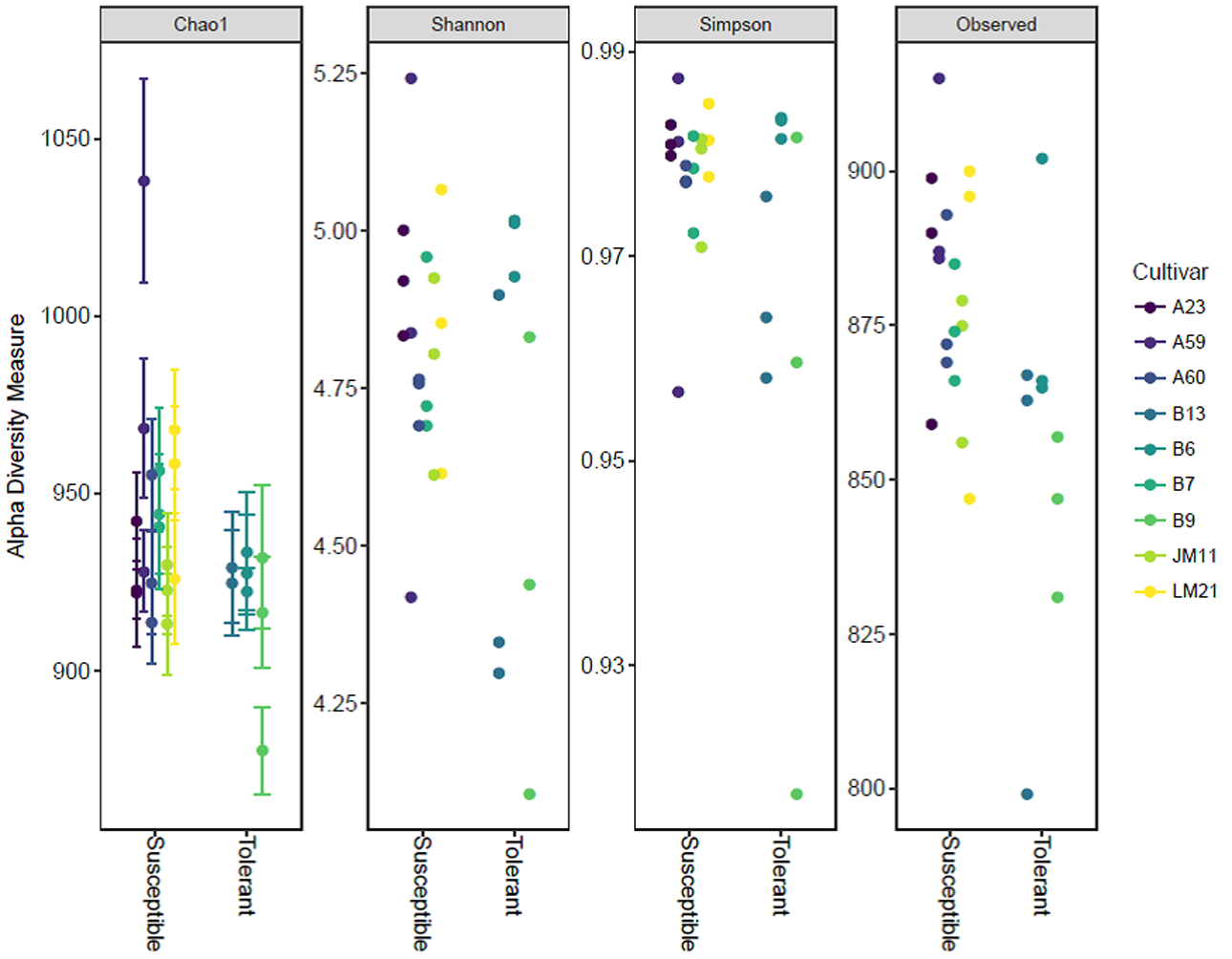

Supplement: Figure S3 — Alpha diversity for endosphere fungi of nine cotton cultivars, classified into wilt tolerant or susceptible groups. Permutation ANOVA was applied to assess the differences in Chao1, Simpson and Shannon indices between the wilt-tolerant and susceptible cultivars. [file Image_3.tif]

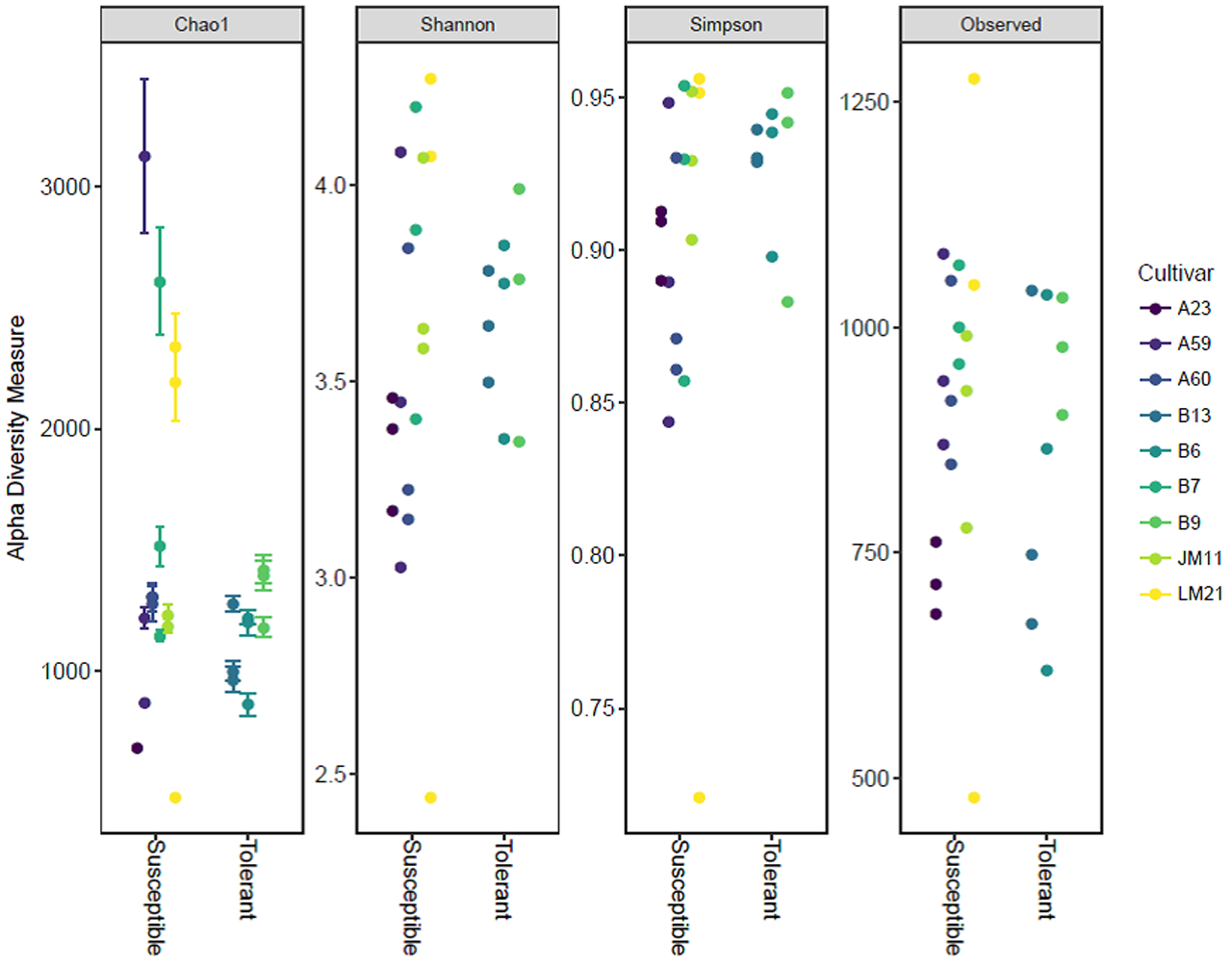

Supplement: Figure S4 — Alpha diversity for rhizosphere fungi of nine cotton cultivars, classified into wilt tolerant or susceptible groups. Permutation ANOVA was applied to assess the differences in Chao1, Simpson and Shannon indices between the wilt-tolerant and susceptible cultivars. [file Image_4.tif]
